# Advances in circulating tumor cells for early detection, prognosis and metastasis reduction in lung cancer

**DOI:** 10.3389/fonc.2024.1411731

**Published:** 2024-06-21

**Authors:** Xiaochen Wang, Lu Bai, Linghui Kong, Zhijuan Guo

**Affiliations:** ^1^ Department of Pathology and Pathophysiology, Inner Mongolia Medical University, Hohhot, Inner Mongolia, China; ^2^ Department of Pathology, Cancer Hospital Affiliated to Inner Mongolia Medical University / Peking University Cancer Hospital Inner Mongolia Hospital, Hohhot, Inner Mongolia, China

**Keywords:** circulating tumor cells, lung cancer, metastatic cascade, early detection, prognosis, targeted therapies

## Abstract

Globally, lung cancer stands as the leading type of cancer in terms of incidence and is the major source of mortality attributed to cancer. We have outlined the molecular biomarkers for lung cancer that are available clinically. Circulating tumor cells (CTCs) spread from the original location, circulate in the bloodstream, extravasate, and metastasize, forming secondary tumors by invading and establishing a favorable environment. CTC analysis is considered a common liquid biopsy method for lung cancer. We have enumerated both *in vivo* and ex vivo techniques for CTC separation and enrichment, examined the advantages and limitations of these methods, and also discussed the detection of CTCs in other bodily fluids. We have evaluated the value of CTCs, as well as CTCs in conjunction with other biomarkers, for their utility in the early detection and prognostic assessment of patients with lung cancer. CTCs engage with diverse cells of the metastatic process, interfering with the interaction between CTCs and various cells in metastasis, potentially halting metastasis and enhancing patient prognosis.

## 1 Introduction

Globally, lung cancer tops the list of causes for cancer fatalities, accounting for approximately 1.8 million deaths attributed to the disease in the year 2020 (1, 2). Lung cancer ranks as the second most commonly identified form of cancer, with nearly 2.2 million new instances annually (1). The World Health Organization (WHO) divides lung cancers into two main categories: non-small cell lung carcinoma (NSCLC), which accounts for approximately 80–85% of all lung cancer diagnoses, and small cell lung carcinoma (SCLC), making up the remaining 15% of cases (3, 4). NSCLC is further broken down into subtypes: adenocarcinoma (LUAD), squamous cell carcinoma (LUSC), and large cell carcinoma (LCC). For both NSCLC and SCLC, especially in their metastatic forms, the prognosis is generally poor, with a five-year survival rate of approximately 4% (5).Constrained by scarce early diagnostic options and genetic tumor diversity, the median survival time for SCLC patients is around 7 to 12 months (6, 7). The IASLC staging data show median survival times of 95 months for stage IA, 75 months for IB, 44 months for IIA, 29 months for IIB, and 19 months for IIIA lung cancer, underscoring the importance of early diagnosis and treatment (8).

**Figure 1:**
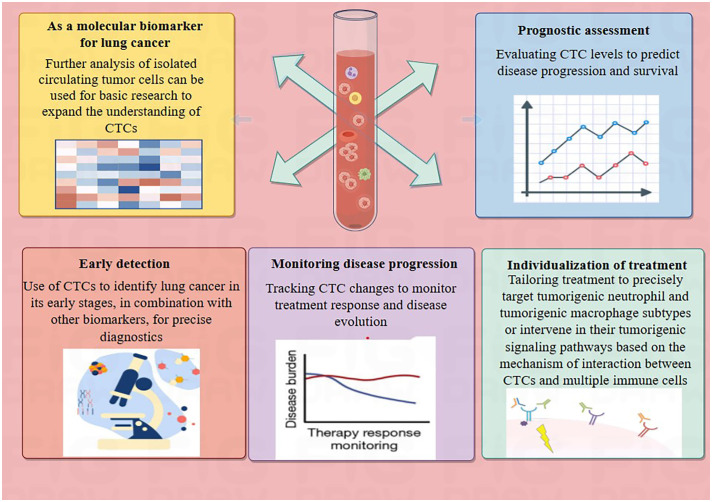
Potential application of CTCs in lung cancer. The summary outlines the potential applications of CTCs in lung cancer patients, which include serving as molecular biomarkers for lung cancer, assessing prognosis, early cancer detection, monitoring disease progression, and facilitating personalized treatment.

Circulating tumor cells (CTCs) represent the initial phase of hematogenous tumor metastasis, with a half-life that varies between 1 to 2.4 hours (9). CTCs are cancer biomarkers that hold promise for early detection. CTCs may arise from either the primary cancer site or from secondary locations where metastasis has occurred and more comprehensively represent a heterogeneous tumor cell population than tissue biopsies (10). CTC isolation and enrichment techniques have advanced from physical properties-based methods (11) to immunomagnetic bead methods targeting specific CTC surface antigens (CK/EpCAM, CD45-) (12), and now to microarray technology (13). The excellent application prospects of CTC in early diagnosis (14, 15), prognostic assessment (16, 17), and precision therapy (18, 19) have been determined. CTCs’ persistence is a significant factor contributing to cancer recurrence and treatment failure across various cancer types (20). Therefore, eliminating CTC or blocking its metastatic process is critical for cancer patients at risk of metastasis. A comprehensive grasp of the biological properties of CTCs and the relationships they have with the microenvironments of the blood circulation and the organs they target can facilitate the early forecasting of metastatic progression and bolster the delivery of clinical treatment strategies (21) (**Figure 1**).

## 2 Overview of clinically available molecular biomarkers for lung cancer

Global standards have outlined prognostic biomarkers essential in the field of chest tumors. According to the ESMO’s ESCAT framework (ESMO Scale for Clinical Actionability of molecular Targets), biomarkers such as ALK, BRAF, EGFR, MET, NTRK, RET, and ROS1 are ranked as Class I (**Table 1**) (22). Both FDA-approved diagnostic tests and lab-created platforms are employed to evaluate these markers (23). The NCCN advises the examination of these significant prognostic biomarkers for patients diagnosed with advanced NSCLC prior to starting therapy, as the selection of appropriate targeted or immunological treatments can be guided by the biomarker test outcomes (23).

**Table 1:** A List of genomic alterations level I/II/III according to ESCAT in advanced NSCLC.

Gene	Alteration	Prevalence	ESCAT	Ref
*EGFR*	Common mutations (Del19, L858R)	15% (50%–60% Asian)	IA	(22)
	Acquired T790M exon 20	60% of EGFR mutant NSCLC	IA	
	Uncommon EGFR mutations(G719X in exon 18, L861Q in exon 21, S768I in exon 20)	10%	IB	
	Exon 20 insertions	2%	IIB	
*ALK*	Fusions (mutations as mechanism of resistance)	5%	IA	
*MET*	Mutations *ex 14 skipping*	3%	IB	
	Focal amplifications (acquired resistance on EGFR TKI in *EGFR*-mutant tumors)	3%	IIB	
*BRAF* ^V600E^	Mutations	2%	IB	
*ROS1*	Fusions (mutations as mechanism of resistance)	1%–2%	IB	
*NTRK*	Fusions	0.23%–3%	IC	
*RET*	Fusions	1%–2%	IC	
KRASG12C	Mutations	12%	IIB	
ERBB2	Hotspot mutations Amplifications	2%–5%	IIB	
*BRCA 1/2*	Mutations	1.2%	IIIA	
*PIK3CA*	Hotspot mutations	1.2%–7%	IIIA	
*NRG1*	Fusions	1.7%	IIIB	

The EGFR gene, encoding a receptor tyrosine kinase, often features deletions within exon 19, the L858R mutation in exon 21, and is also marked by the T790M mutation in exon 20. Phase III clinical trials have demonstrated that the use of EGFR TKIs can enhance survival rates in patients with NSCLC harboring EGFR mutations (24–26). These particular EGFR mutations are classified at the top tier of the ESCAT scale. EGFR-targeted TKIs are now the preferred initial treatment for NSCLC with EGFR mutations. Advances in EGFR-targeted therapies have extended the OS for patients with these mutations beyond three years (25). Yet, resistance limits the long-term efficacy of these drugs, leading to the development of next-gen EGFR inhibitors like afatinib, osimertinib, dacomitinib, and rociletinib, with osimertinib outperforming standard chemotherapy in ORR and PFS (26). The ALK fusion gene, also highly ranked in ESCAT, has shown improved patient outcomes in clinical trials involving ALK inhibitors for ALK-rearranged NSCLC (27–29). Additional genetic alterations include MET exon 14 skipping, BRAF^V600E^ mutations, and ROS1 fusions (30). Phase I/II studies have reported significant ORR and clinical benefits in patients with MET exon 14 mutations treated with crizotinib, those with BRAF^V600E^ mutations treated with dabrafenib-trametinib, and ROS1 fusion patients treated with crizotinib and entrectinib (31–34). Although NTRK gene fusions are rare in a spectrum of cancers, TRK inhibitors such as larotrectinib and entrectinib have demonstrated enduring remissions in NTRK fusion-positive tumors, including lung cancers (35, 36). While not yet systematically assessed in clinical practice, emerging biomarkers such as NRG1 fusions, PIK3CA mutations, BRCA1/2 mutations, non-V600 MAP2K1 mutations, and HER2 amplifications hold promise for identifying novel therapeutic targets (37).

The expression of PD-L1 serves as a crucial marker in immuno-oncology. It is increasingly present in various cancers, including NSCLC, and correlates with an unfavorable outcome and reduced survival times for patients (38). While PD-L1 levels are commonly utilized to predict response to immunotherapy, they are not always a reliable indicator, as some patients with minimal PD-L1 expression may benefit from such treatments, whereas others with high expression may not (23). The KEYNOTE-042 clinical study endorses pembrolizumab for first-line treatment in cases of advanced or metastatic ncccon-small cell lung cancer (NSCLC) (39). Furthermore, the IMpower110 trial, carried out in 2020, suggests that atezolizumab may serve as an first-line treatment for patients with NSCLC exhibiting PD-L1 expression, regardless of their tumor’s histological subtype (40).

## 3 Transfer process of CTCs

Metastasis is the main cause of death in solid tumor cases. Cells detach from the primary or secondary tumors enter the bloodstream, spreading to organs like the lungs, liver, brain, and lymph nodes. These circulating cells are termed circulating tumor cells (CTC).To facilitate distant metastasis, CTCs need to separate from the primary tumor site and undergo a transformation from an epithelial to a mesenchymal state. This shift, termed Epithelial-to-Mesenchymal Transition (EMT), is essential for the metastatic spread of aggressive cancers (41). CTCs gain the ability to detach from the basement membrane after undergoing Epithelial-Mesenchymal Transition (EMT). During the EMT process, tumor cells lose epithelial phenotypes such as connection with the basement membrane, reduce intercellular adhesion, and acquire epithelial (E), mesenchymal (M), or epithelial/mesenchymal (E/M) hybrid phenotypes (42). Human lung cancer cells with E/M hybrid phenotype are more capable of proliferating and invading the surrounding environment (43). An NSCLC cohort study (21) confirmed that E/M-CTCs are meaningful in forecasting unfavorable patient prognosis and metastasis (44). In a study utilizing bioinformatics analysis with seven gene microarrays, Guan et al. noted that CTCs, when compared to the originating tumor, predominantly exhibit alterations in cellular adhesion, the process of EMT, and apoptotic pathways (45). Further research has indicated that fluid shear stress can trigger EMT in CTCs via the JNK signaling cascade in breast cancer, reinforcing the correlation between an increase in EMT within CTCs and reduced patient survival rates (46).

EMT may be affected by various cytokines and tumor microenvironmental stimuli, transforming, growth factor beta (TGF-β) is an effective induction factor of EMT development (47–49). TGF-β binding to its receptor can activate the PI3K/AKT and RAS/MAPK signaling pathways, result the suppression of genes related to epithelial phenotype and the activation of genes related to mesenchymal phenotype (49). The occurrence of EMT was also affected by the metabolic condition of CTCs (47). Mice on a high-fat diet exhibit elevated release of CTC and excessive incorporation of free fatty acids into cell membranes, promoting increased tissue invasion and lung metastasis (50). CTCs with elevated PGK1 and G6PD levels enhance glucose metabolism, increasing EMT incidence and invasiveness (51).

Tumor cells that have shed enter the bloodstream through the process of intravasation, existing sparsely as CTCs—typically numbering only a few to several hundred per 10 mL of blood. In the circulation, cancer cells must escape blood flow shear stress and the immune system to avoid destruction by shear forces and antitumor immune cells, and the formation of platelet aggregates around CTCs blocks NK cells and circulatory shear forces (52). CTCs may facilitate metastasis by interacting with leukocytes, platelets, immune cells, and cancer-associated fibroblasts in the blood to create heterogeneous clusters, which raises the potential for the metastatic settlement of CTCs (53). Despite the infrequency of CTC clusters in the peripheral blood, these clusters are more stable within the bloodstream than solitary CTCs and are associated with a higher propensity for metastasis (54). Moreover, patients who lack these clusters at the baseline have markedly improved PFS and OS compared to those with detectable CTC clusters at the same time point (55).

**Figure 2:**
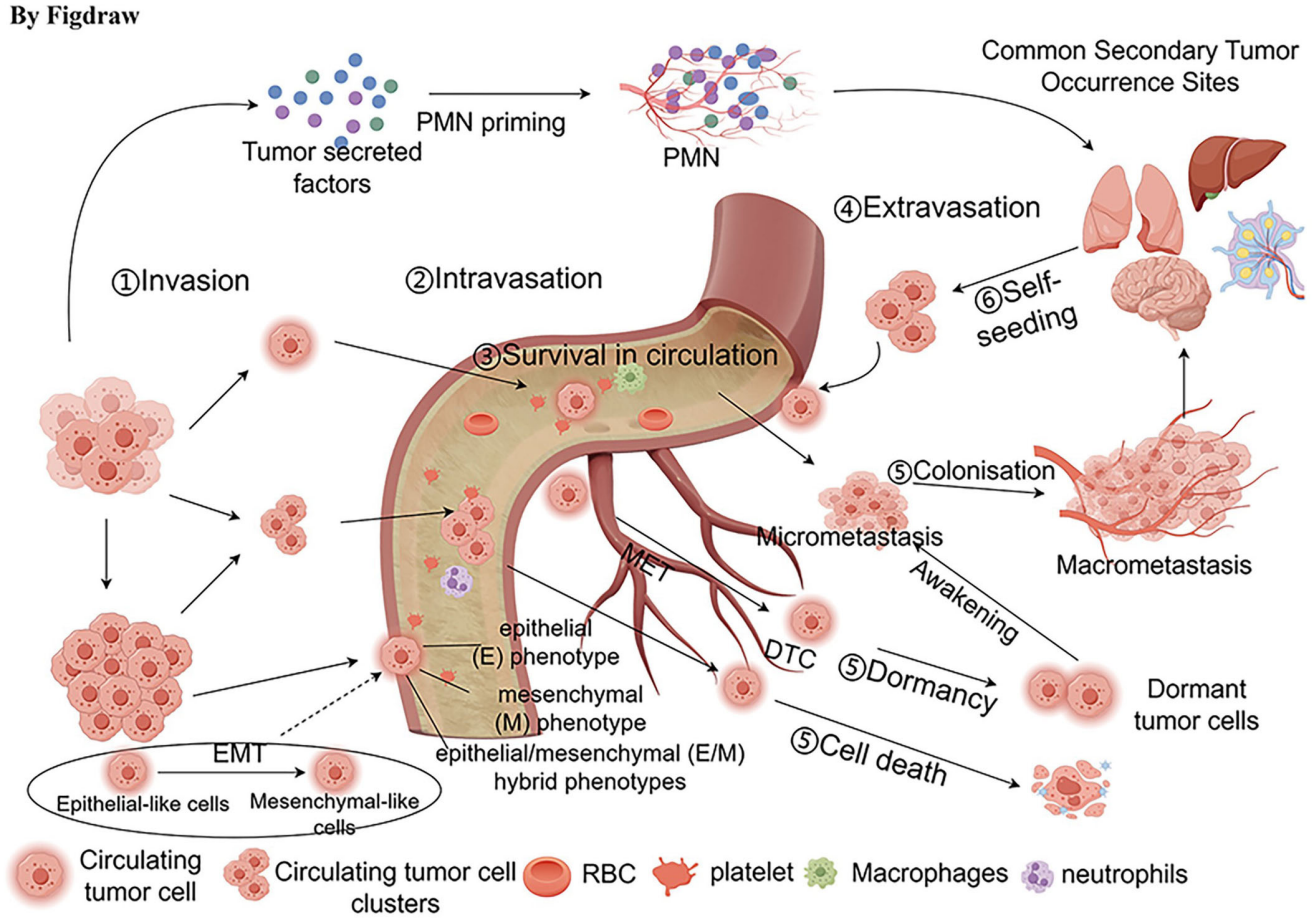
Transfer process of CTCs. The process of metastasis includes the detachment of CTCs from the primary tumor, undergoing epithelial-mesenchymal transition (EMT), intravasation into the bloodstream, survival in the circulation, interaction with various cells, and extravasation out of the circulation. Some disseminated tumor cells (DTCs) can enter a dormant state, existing as micrometastatic lesions or as individual tumor cells. Additionally, some DTCs can migrate to new metastatic sites, such as the liver, lungs, brain, and lymph nodes.

Upon survival, CTCs preferentially extravasate from the circulation into the pre-metastatic niche (PMN), experience mesenchymal to epithelial transition(MET) and move to new metastatic sites as disseminated tumor cells (DTCs) (52, 56). PMN is formed before the primary tumor reaches the DTC, and the original tumor modifies the microenvironment by releasing growth factors, exosomes, and extracellular matrix remodeling in order to facilitate tumor metastasis. The destiny of DTCs is influenced by both inherent and external elements within the cellular and tumor microenvironment. A portion of DTCs become dormant and can be present as micrometastases or individual tumor cells (57). Research has found that in 36% of patients, CTCs could be identified up to 8 to 22 years post-mastectomy without any signs of advancing tumor growth. This occurrence could be linked to the absence of completing the ultimate phase of metastasis, potentially regulated by the equilibrium between cell apoptosis and cell division, thus preserving a dormant state (58). Additional factors influencing the dormancy of micrometastases include a lack of immune surveillance, impact from the tumor microenvironment, and therapeutic stress (59). Micrometastases can also evolve into macrometastases. Furthermore, CTCs have the ability to migrate back to the primary tumor site and reinoculate. This phenomenon was demonstrated by Kim et al. and called “tumor self-seeding”, indicating that the migration of CTCs is multidirectional and can involve movement towards the primary tumor site (60) (**Figure 2**).

## 4 CTCs enrichment and separation technology

Enrichment of CTCs predominantly occurs during the intravasation phase as they enter the circulatory system, which typically refers to the bloodstream. EMT is the basis for the migration, anti-apoptosis, infiltration, and metastasis of CTCs (61).CTC detection is difficult because of their ability to undergo EMT resulting in a lack of surface expression of epithelial markers (20). CTCs alter their epithelial/mesenchymal phenotype to better suit the local microenvironment during both phases (62). EpCAM and vimentin are ubiquitous in all tumor cells and can be markers for identifying any CTC (63). Enrichment and separation of CTCs often use EpCAM and the exclusionary biomarker CD45. Granulocytes often express low levels of CD45 and high levels of CD15, making them susceptible to being misidentified as CTCs (64). Utilizing CD15 antibodies in conjunction with highly specific CD45 antibodies might enhance the accuracy of CTC detection and eradicating false positives by double exclusion (64). However, transitioning from laboratory CTC enrichment and separation to clinical practice remains challenging, and sensitive and reliable CTC capture methods are essential for CTC research. Here, we outline the significantly advanced technologies in this area (**Table 2**).

**Table 2:** CTC enrichment and separation technology.

Method	Advantage	Disadvantages	Methods		Ref
Enrichment and separation *in vivo*	Highly sensitive, can detect higher amounts of ctc, suitable for early tumor diagnosis	Complex procedures, invasive, biosafety issues, expensive associated costs	*In vivo* capture of CTCs based on epithelial cell surface adhesion molecule (EpCAM) antibody-modified intravenous indwelling needle blood transfusion		(65)
			3-D CTC-Net probe for *in vivo* capture of CTCs		(66)
			GILUPI cell collector captures CTC directly into peripheral veins		(67)
			Three-dimensional magnetic chip with extracorporeal circulation		(68)
*In vitro* separation and enrichment methods based on physical properties	Detection is fast and can detect CTC with EpCAM negative and unaffected by marker expression	It has low detection efficiency, poor CTC purity, and captured a large number of non-CTC cells. Smaller or inconsistent density CTC may be missed, and the pressure and shear forces used during separation may damage the cells	Cell size-based separation and enrichment methods	ISET DEF SSA-MOA	(69) (70) (71)
			Density-based separation and enrichment methods	Ficoll-Paque OncoQuick	(72)
			Dielectrophoresis	ApoStream	(73)
			Microfluidic separation of CTCs based on their physical properties	Parsortix, ClearCell FX VTX-1	(74) (75) (76)
*In vitro* separation and enrichment methods based on biological properties: positive enrichment	Provides high enrichment efficiency and product purity	CTCs that have lost EpCAM expression cannot be detected	CellSearch Adnatest Metal-organic framework materials		(77) (78) (79)
*In vitro* separation and enrichment methods based on biological properties: negative enrichment	High cell viability; avoid loss of EpCAM-negative CTCs	A large number of non-target cells are isolated.	RosetteSep		(80)

**Figure 3:**
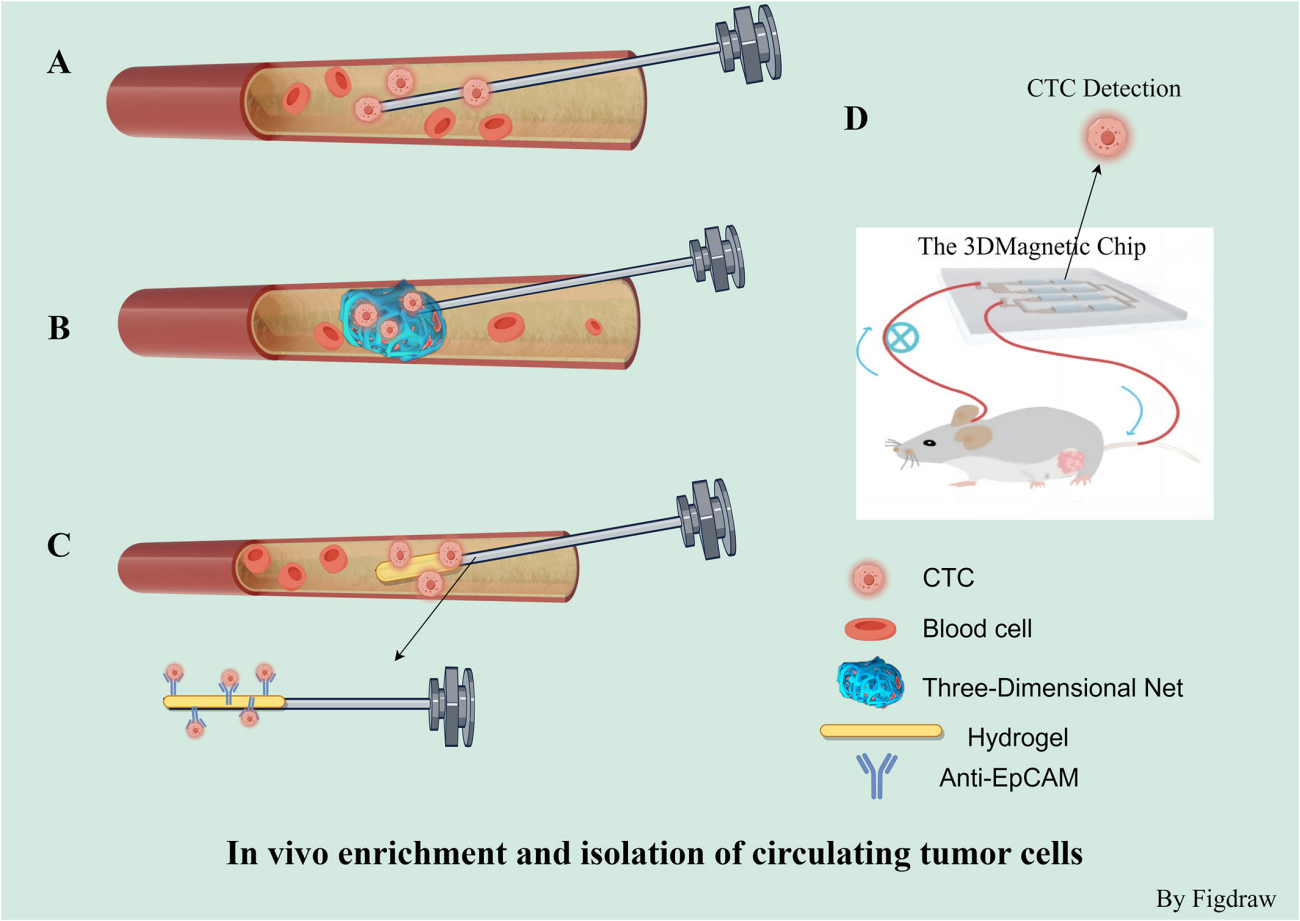
*In vivo* enrichment and isolation of circulating tumor cells. **(A)** *In vivo* capture of CTCs based on epithelial cell surface adhesion molecule (EpCAM) antibody-modified intravenous indwelling needle blood transfusion. **(B)** 3-D CTC-Net probe for *in vivo* capture of CTCs. **(C)** GILUPI cell collector Is a medical stainless steel wire with a 2 cm long functional domain coated with EpCAM antibodies and a hydrogel layer. **(D)** 3D magnetic chip for extracorporeal can be used for *in vivo* detection and real-time monitoring of CTC.

Several methods have been created to separate CTCs by utilizing their distinct physical and biological characteristics. Current enrichment and separation techniques can be categorized *in vivo* and *in vitro*. Contrasting with *in vitro* assays for CTCs, *in vivo* methods can detect a higher quantity of CTCs and are capable of identifying these cells in the bloodstream during the initial phases of tumor development. These methods are highly sensitive and thus well-suited for early cancer detection. Nonetheless, the intricacy of the procedures involved, along with biosafety issues and the associated expenses, have impeded their widespread use in clinical settings. *In vivo* methods encompass the use of EpCAM antibody-modified intravenous indwelling needle for capturing CTCs during blood transfusion (65), the three-dimensional CTC-Net probe is engineered for the collection of CTCs within blood vessels (66), and the GILUPI CellCollector (67). Jiao and colleagues have also described a 3D magnetic chip for ex vivo circulation, which can be utilized for *in vivo* detection and real-time monitoring of CTCs (68). To assess the feasibility of using the CellCollector for early lung cancer detection, a study involving 64 participants was conducted. Findings indicated that the CellCollector effectively distinguished between malignant and benign lung nodules, highlighting its potential for early lung cancer identification (67) (**Figure 3**).

Utilizing the physical properties of cells, ex vivo separation methods can successfully isolate CTCs without relying on specific markers, which is high efficient for capture (81). However, these methods might not be as effective in detection and could accidentally collect many non-CTC cells. There’s also a chance that smaller or inconsistently dense CTCs might be missed. Additionally, the forces used in the separation process could possibly harm the cells. The ISET system leverages differences in cell size to isolate epithelial tumor cells, providing a straightforward and economically feasible method for enriching fixed CTCs (69). Additional size-based detection methodologies include DEF (70) and SSA-MOA (71). Density gradient centrifugation is a method used to concentrate CTCs. Various commercial tools, kits, and reagents exist for this technique, such as the Ficoll-Hypaque and OncoQuick systems. These devices help separate CTCs through centrifugation in a medium containing a porous partition and an appropriate density gradient (72). The ApoStream system captures CTCs using dielectrophoresis field-flow fractionation (DEP-FFF) within a microfluidic chamber (73). In recent years, a number of microfluidic chip-based cell separation systems have been introduced, including the Parsortix (74), ClearCell FX (75), and VTX-1 (76). For example, the Parsortix is a microfluidic device that doesn’t rely on specific epitopes. It uses tiny stepped structures that get progressively narrower to target rare cells based on size and deformability (74). The ClearCell FX system has shown it can recover more than 60% of the NCI-H1650 lung cancer cells added to blood samples (75). Zhu et al. have introduced a novel approach to address the low purity and potential cell damage associated with ex vivo CTC separation techniques. By adhering engineered RBCs to CTCs to create CTC-RBC complexes, they achieved over 90% capture efficiency and over 75% purity (82). Additionally, using autologous RBCs bound to CTCs (CC-RBCs), they leveraged differences in optical properties to separate modified CTCs in an optofluidic system with a laser, resulting in a purity above 92% and a recovery rate exceeding 90%, while maintaining the CTCs’ membrane integrity and function (83).

**Figure 4:**
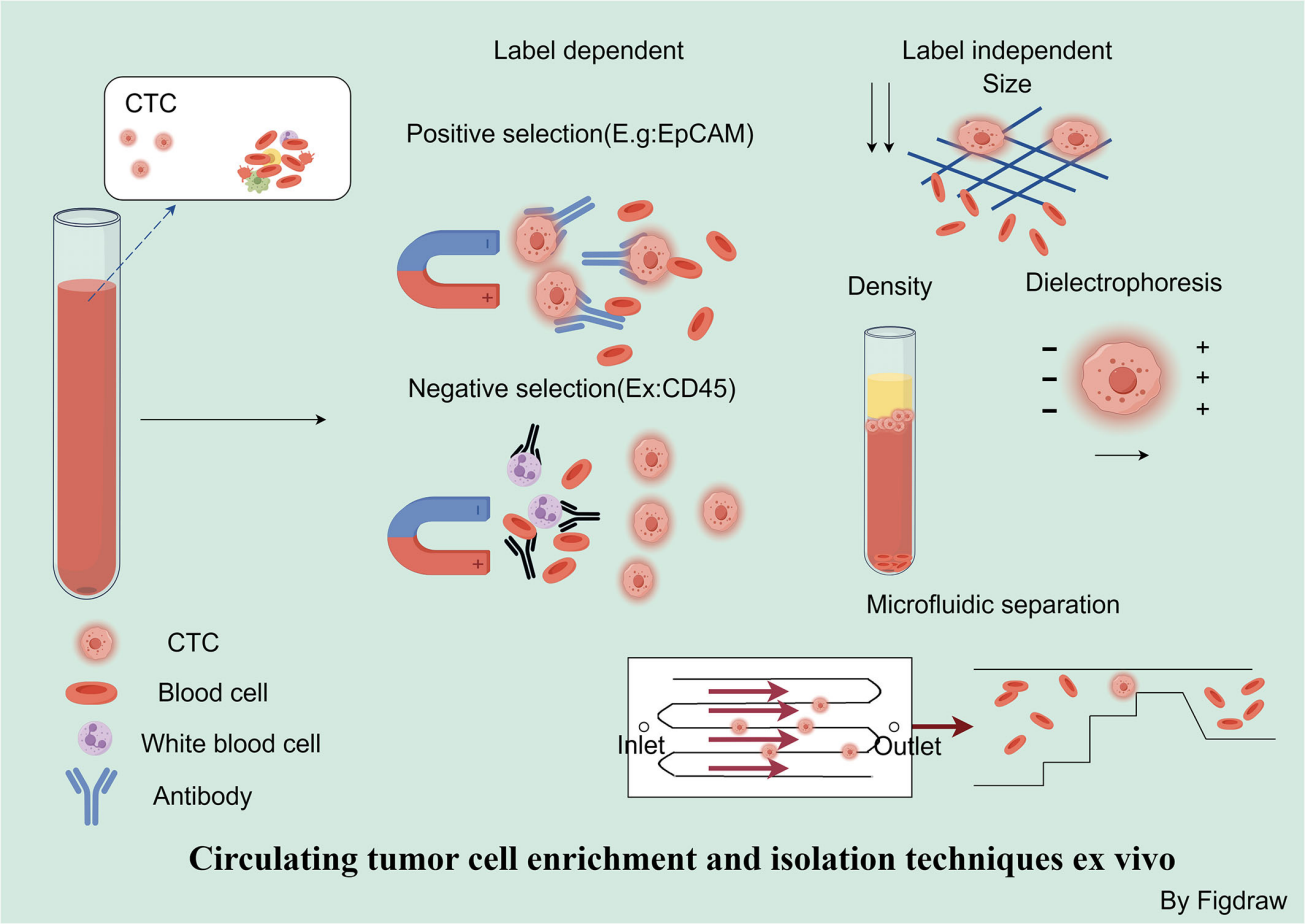
Circulating tumor cell enrichment and isolation techniques ex vivo. Strategies for the separation and enrichment of CTCs can be divided into marker-dependent techniques based on their biological characteristics and marker-independent techniques based on physical properties. In marker-dependent techniques, immunomagnetic separation utilizes EpCAM antibodies for positive selection, while negative selection depletes white blood cells and platelets by recognizing CD45. Marker-independent techniques include size-based, charge-based, and density-based separation methods, as well as microfluidic separation based on physical properties.

Ex vivo separation and enrichment methods based on biological properties include both positive and negative enrichment. The CellSearch system, which includes immunomagnetic separation based on EpCAM, followed by immunofluorescent imaging and detection of epithelial-derived CTCs, was the first to receive FDA approval for clinical use. However, it may not capture CTCs that are undergoing EMT (77, 84). Within the AdnaTest detection system, a combination of immunomagnetic separation with multiplex reverse transcription polymerase chain reaction (RT-PCR) is implemented to segregate and detect CTCs from blood. This approach permits the profiling of gene expression specific to certain tumor-linked markers (78). Metal-organic framework materials that can respond to changes in pH values and are modified with anti-EpCAM antibodies on the surface of MIL-100 can be utilized for the capture of CTCs. These materials are capable of auto-degrading in an acidic environment, which enables the self-release of CTCs (79). However, CTCs that have undergone EMT are negative for EpCAM and thus cannot be isolated by assays specific for anti-EpCAM (85). In such cases, negative enrichment represents a more ideal approach for isolating CTCs. RosetteSep represents a negative enrichment technique for CTCs, which aids in obtaining viable CTCs and clusters. This is achieved through centrifugation to eliminate CD45-positive cells that have bound to antibodies in a tetramer form and subsequently settled within a Ficoll-Paque density gradient (80). Positive selection offers the advantage of providing high enrichment efficiency and product purity, but it has the drawback of being unable to detect CTCs that have lost EpCAM expression. Conversely, negative selection methods have the advantage of capturing all possible CTC populations but also result in the isolation of a large number of non-target cells (**Figure 4**).

In addition to being present in the bloodstream, CTCs can also be identified within other body fluids, including lymphatic and cerebrospinal fluids. Utilizing *in vivo* photoacoustic and fluorescent flow cytometry techniques, researchers have detected lymphatic tumor cells (L-CTCs) in preclinical mouse models for melanoma and breast cancer. This approach allows for the recognition and measurement of the initial L-CTCs during the pre-metastatic stage (86). The challenge with L-CTCs stems from the delicate structures, which have low pressure and cell concentration, requiring the use of lymphangiography for their detection and localization, a procedure not routinely performed in clinical practice (86). Multispectral photoacoustic flow cytometry (PAFC) allows for *in vivo* molecular detection in cerebrospinal fluid. The simultaneous detection of CTCs in blood, cerebrospinal fluid, and sentinel lymph nodes at the same point in tumor progression suggests that CTCs may disseminate through three types of body fluids (87). Akshal also modified the CellSearch system for the detection of cerebrospinal fluid tumor cells (CSFTCs) by spiking cerebrospinal fluid samples into normal human blood for testing (88). Research on the detection and analysis of CTCs in other bodily fluids is more scarce and challenging. Overcoming the technical limitations of detection in cancer research may pave the way for new avenues of CTC detection and expand our understanding of the role of CTCs in the development of metastasis.

## 5 Application of CTC in clinical early cancer detection and prognosis assessment

### 5.1 CTCs for early cancer detection

Because CTCs were thought to be present only in the late stages of the disease and detection methods were subject to technical limitations, initial research on CTCs did not explore their role in early cancer detection (89). Evidence suggests that cancer cells can swiftly invade local tissues and spread within hours (90). Multiple studies have assessed the capability of CTCs for detecting cancer by analyzing samples from individuals already diagnosed with cancer. In research, CTCs were found in 49% of patients with stage I NSCLC, a percentage similar to those with stage II to IV illness (91). CTC counts in tumor-draining pulmonary veins and peripheral blood were analyzed using CellSearch in 30 patients diagnosed with stage I to IIIA NSCLC. The CTC values of peripheral veins and PV are independent risk factors for lung cancer recurrence. The count of PV CTC is also an independent risk factor for death (92). In a study of 168 patients with COPD using ISET technology, five patients with detectable CTC were subsequently diagnosed with NSCLC within 1–4 years by annual CT screening (14). CTC detection can be combined with other detections to avoid false diagnoses and more accurately study the role of CTC in the early detection of lung cancer.

### 5.2 CTCs combined with other biomarkers for early cancer detection

Cancer diagnostics with biomarkers hold promising potential for early detection and ongoing disease monitoring (93). High-throughput targeted DNA methylation sequencing of circulating tumor DNA (ctDNA) (94) and miRNA (95) has demonstrated promise as early cancer detection methods. Combining these biomarkers with the CTC test may improve the sensitivity and specificity of the test. In a group of 111 patients, the diagnostic sensitivity for ctDNA and CTC detection was 72.7% and 65.7%, respectively. The sensitivity of the combined CTC/ctDNA assay (95.0%) was significantly higher than that of CTC alone (p<0.001), ctDNA alone (p<0.001), or conventional tumor markers (p<0.001) (96). The combination of ctDNA and CTC measurements may help detect primary lung cancer (96). CTC and circulating tumor microemboli (CTM) were jointly detected in 30 early-stage NSCLC patients, and multivariate analysis validated the predictive significance of detecting both CTM and CTC together (92).

Aside from these circulating biomarkers, various methods have been utilized in clinical settings for early cancer diagnosis. Radiography is frequently utilized for cancer diagnosis and population screening for lung cancer (97). CTCs have similar diagnostic accuracy to various imaging modalities with higher specificity and better prognostic accuracy in lung cancer patients (98). Current research emphasizes proven techniques to detect proliferating CTCs, assess cell proliferation, and create predictive models using non-invasive screening tests (99). In summary, various methods combined with CTC detection have attained cutting-edge outcomes in cancer prevention, treatment and personal health status assessment.

### 5.3 The prognostic value of CTCs

The study proposes that CTC, in conjunction with ctDNA, might serve as a supplementary biomarker for early detection of therapy response (100, 101). CTCs may also be utilized with tissue-based identification of EGFR mutations in lung cancer to assist in precisely treating such alterations when tissue biopsy samples are unavailable for testing (102). A recent study explored the relationship between clinical staging (TNM) and the detection of CTCs. The data suggest that the mean proportion of mesenchymal CTCs increases in advanced lung cancer compared to early stages, and CTCs exhibiting a mesenchymal phenotype are strongly associated with metastasis to the lymph nodes or blood vessels (103). Phenotypic analysis of CTCs may also have clinical utility in stratifying breast cancer patients for HER2-targeted therapy, proving that continuous monitoring of CTC numbers and subtypes can help clinically determine the development status and prognosis of tumor diseases (104). Cluster analysis can be performed using phenotypic similarities of different CTC subpopulations to identify potential biomarkers indicative of organ-specific metastatic trends (105).

Lin et al. quantified the CTCs in the peripheral blood of stage IV NSCLC patients before and after NK cell immunotherapy. This study offers a valuable benchmark for monitoring any changes in the efficacy of NK cell therapy (106). The CTC counts, and the percentage of CTC-positive cases were lower than before radiochemotherapy in 58 patients with advanced NSCLC treated with simultaneous radiochemotherapy (107). Positive CTCs after radiochemotherapy and increased CTCs during radiochemotherapy are associated with decreased PFS (107). Purcell et al. analyzed CTCs at six-time points in 26 patients throughout chemoradiotherapy and immune checkpoint inhibitor therapy. A significant reduction in CTCs following therapy was observed, with a more significant decrease in CTCs predicting longer progression-free survival (108). CTC counts are also valuable during cancer treatment. During treatment, the number of CTCs was positively correlated with various clinicopathologic variables (109). CTC counts and proliferative capacity showed a high correlation with the prognosis of lung adenocarcinoma (110) and primary unspecified cancers (111).

## 6 Anti-metastatic therapy based on the interaction of CTC with different components of the tumor microenvironment

### 6.1 Disruption of CTC-platelet interactions

CTCs interact with platelets to help CTCs escape the immune response in the circulation and utilize them to improve their adhesion and promote metastasis (112). Platelets interact with CTCs via adhesion proteins such as fibronectin and integrins to facilitate cluster formation, and platelet-encapsulated CTCs are more likely to escape immune system surveillance (113). At the same time, platelets can prevent NK cell-mediated cytolytic attacks by creating a fibrinogen-rich barrier and transferring MHC-I to CTCs (114). Platelets in NSCLC patients also express PD-L1, which inhibits CD4 and CD8 T cells and contributes to tumor immune escape (115).Tumor cell-induced platelet aggregation (TCIPA) is another factor that impacts malignancy in CTC-platelet interactions. The specific mechanism of platelet aggregation by malignant tumor cells depends on the type of tumor cells (116). Certain cancer cell lines, including breast cancer (117), lung cancer (118), pancreatic ductal adenocarcinoma (119), and HT-1080 fibrosarcoma (118), can generate ADP and induce TCIPA. In addition, cancer cells can produce thrombin, which may influence tumor growth, angiogenesis, and metastasis (120). Cathepsin K, like ADP and thrombin can promote interaction between platelets and cancer cells, induce TCIPA and promote metastasis (121). The research results of Alba et al. demonstrated for the first time that platelets not only indirectly activate pathways related to the microenvironment, but also efficiently convey lipids, proteins and RNA via various mechanisms to educate tumor cells. The interaction with platelets may lead to tumor cells and CTCs adopting highly dynamic and aggressive traits, which can involve EMT, stem cell-like phenotype, and elevated proliferative capacities (122).

Targeting platelet/coagulation cascade interactions may provide a pathway for CTC destruction and inhibit disease progression. In a study by Li et al., platelets were genetically modified to express TNF-related apoptosis-inducing ligand (TRAIL) on the cell surface, which was shown to eliminate tumor cells *in vitro* and reduce metastasis (123). Ortiz-Otero et al. transferred TRAIL to platelets by interaction with the von Willebrand factor (vWF). TRAIL-coated platelets kill CTCs in blood, reducing cancer metastasis (124). Both approaches have achieved promising results in effectively targeting and killing CTCs *in vivo*. Using the principle that CTCs rely on platelet assistance for survival, Anne et al. described that detergent-treated drug-free “platelet bait” and inert platelets showed negative effects in both metastatic and thrombosis *in vivo (* 125). Activation of platelet P2Y12 receptors triggers the secretion of more ADP, ATP, and calcium from dense granules, enhancing the activation of platelets triggered by ADP (126). Ticagrelor dramatically decreases platelet-cancer cell interactions and metastasis by inhibiting platelet P2Y12 receptors (127). P-selectin (128), CLEC-2 (129), protease-activated receptor 1 (PAR-1) (130), and integrins (including αIIbβ3, αvβ3, α6β1, and α2β1) (131) can all serve as key receptors that mediate the binding and activation of platelets and tumor cells, plays a role in tumor progression and has the potential to serve as an anti-cancer target.

### 6.2 Disruption of neutrophil-CTC interactions

Neutrophils are the most abundant immune cells and among the initial immune cells contacted by CTCs upon entering the blood circulation (132). In recent years, increasing evidence suggests that neutrophils can interact with CTCs as cancer modulators. Neutrophils in tumors are divided into subpopulations that are polarized into N1: antitumor neutrophils and N2: protumorigenic neutrophils, which may have opposing effects on each stage of the tumor, including initiation, proliferation, metastasis, and immunosuppression (133–135). The literature describes two possible mechanisms in which neutrophils and CTC might interact (1): Through direct interaction to form clusters, CTC-WBC clusters can be transported throughout the body via neutrophils by various mechanisms (136), and (2) neutrophils can aid tumor metastasis by assembling nucleated DNA-containing neutrophil extracellular traps (NETs) to effectively capture CTC-WBC clusters and promote cancer progression (137). A meta-analysis indicates that the presence of CTC-WBC clusters is consistently linked with poor prognosis (138). Neutrophils play an important role in promoting CTC metastasis and the advancement of cancer.

Surface-expressed ligands in many CTCs bind to E-selectin (ES) expressed on endothelial cells, triggering tumor cell autophagy induced by the death receptor TRAIL (139). Leukocytes loaded with ES and TRAIL liposomes can enhance the endocytosis of CTCs, decrease the number of CTCs, and inhibit metastasis (140). Research by Zhang et al. found that using neutrophil chemotaxis to develop drugs targeting CTCs using neutrophils as carriers can effectively reduce the number of lung metastases in mice (141). Targeting the combination of CTCs and neutrophils to inhibit cancer metastasis may also be a potential anti-metastasis therapeutic strategy. Szczerbas et al. discovered that VCAM1 can inhibit the formation of CTC-neutrophil clusters (136). Cellular connections between CTCs and neutrophils detach upon treatment with VCAM1 monoclonal antibody (142). Activated neutrophil membrane-coated nanoparticles (aNEM NPs) impede with neutrophil recruitment to primary tumors and PMN, prevent neutrophil adhesion to tumor vascular endothelium and CTCs, and dismantle the aggregation of CTCs with neutrophils both *in vitro* and *in vivo (* 143).

### 6.3 Targeting tumor-associated macrophages

Tumor-associated macrophages (TAM) are immune cells in the tumor microenvironment with two broad phenotypes: pro-inflammatory, anti-tumorigenic M1 phenotype and anti-inflammatory, pro-tumorigenic M2 phenotype, which are plastic (144, 145). During advanced stages of tumor, the M2-polarized state can persist for extended durations, promoting angiogenesis, tumor advancement, immune system evasion, and the establishment of immunological tolerance via cytokine release (144, 146–148). Macrophages can interact with the tumor cells through direct contact or by releasing cytokines, degrading the extracellular matrix, and facilitating CTC migration and adhesion to vascular endothelial cells (149). Therefore, targeting TAM has emerged as a strategy to reduce metastasis (150). TAM can also contribute significantly to tumor metastasis by inducing EMT in cancer cells, as exemplified in hepatocellular carcinoma (151), gastric cancer (152), and colorectal cancer (153). Numerous studies have documented the link between TAM and the prognostic outcomes for lung cancer patients, predominantly suggesting that the presence of TAM correlates with an poor prognosis in the course of lung cancer, but adenocarcinoma (154–156) predominates, and there are fewer reports on the relationship between the prognosis of squamous cell carcinoma and neuroendocrine carcinoma (157).

TAMs play an important role in limiting the immune system’s resistance to cancer and are considered potentially powerful targets for cancer immunotherapy (149). Novel therapeutic strategies for targeting TAMs in lung cancer include inhibiting of macrophage recruitment with drugs such as CSF1R antibody (158) and C-X-C motif chemokine ligand 12 (CXCL12) inhibitors. Colony Stimulating Factor 1 Receptor (CSF1R) is a key component found in TAM that influences tumor monocyte recruitment and shapes its function within the TME. It could also be a therapeutic target. Currently, several clinical trials are actively underway (159). Reprogramming TAM to promote M2/M1 switching of TAM (160) aims to force M2 to M1 phenotype switching to interrupt the positive feedback between TAM and CTC (161). Several studies have shown that reprogramming TAM can be facilitated (162–165). A study that suggests selective removal of TAM from TME and enhancement of its antitumor activity might be a promising treatment approach for cancer prevention (166). TAMs can also serve as cell carriers to mediate biomimetic delivery systems to enhance the efficacy of antitumor drugs (167). Exploring the synergistic effects of TAM-targeted therapy in combination with existing therapies, including radiotherapy (168), chemotherapy (158), anti-EGFR therapy (169), and photodynamic therapy (170), the combination achieved satisfactory therapeutic results.

## 7 Discussion

Biomarkers facilitate not just the identification of diseases but also the selection of therapeutic strategies, and tracking of cancer progression and its heterogeneity to combat drug resistance. In lung cancer, these molecular signatures allow for the customization of treatment approaches based on the distinct molecular profile of an individual’s tumor, furthering the field of personalized medicine. Analysis of CTCs can provide a wide range of information for clinical lung cancer management, including early warning, diagnosis, and prognostic assessment. Over the past few decades, there has been a swift progression in the development of early detection techniques, with the application of CTCs in Non-Small Cell Lung Cancer NSCLC showing potential. However, their use has not been implemented in routine clinical practice due to the symptoms of instability in the initial stages of the primary tumor. Developing standardized platforms that enhance both the sensitivity and the specificity for detecting and analyzing CTCs is crucial for accurate diagnosis and personalized treatment. It is important to validate their effects in large patient groups due to the significant impact CTCs have on patient prognosis. Several studies have examined the role of various cell types in the circulation interacting with CTCs to promote tumor progression and inhibiting metastasis by targeting these cells. The development of these therapies offers great potential to improve patient prognosis and halt disease progression, and the use of targeted therapies in combination with other therapies holds promise. However, given the indispensable role of platelets, neutrophils, and macrophages in maintaining normal physiological function and immunity, complete inhibition of these cell populations may carry an increased risk of complications. Therefore, precise targeting of pro-tumorigenic neutrophil and pro-tumorigenic macrophage subtypes or intervention in their pro-tumorigenic signaling pathways becomes a more prudent and beneficial cancer treatment strategy.

## Author contributions

XW: Writing – original draft. LB: Writing – original draft. LK: Writing – review & editing. ZG: Writing – review & editing.
